# An Essential Role of Innate Lymphoid Cells in the Pathophysiology of Graft-vs.-Host Disease

**DOI:** 10.3389/fimmu.2019.01233

**Published:** 2019-06-06

**Authors:** Liang Shao, Shan Pan, Qiu-ping Zhang, Muhammad Jamal, Lu-hua Chen, Qian Yin, Ying-jie Wu, Jie Xiong, Rui-jing Xiao, Yok-lam Kwong, Fu-ling Zhou, Albert K. W. Lie

**Affiliations:** ^1^Department of Hematology, Zhongnan Hospital of Wuhan University, Wuhan, China; ^2^Department of Immunology, School of Basic Medical Sciences, Wuhan University, Wuhan, China; ^3^Department of Medicine, Li Ka Shing Faculty of Medicine, Faculty of Social Sciences, The University of Hong Kong, Hong Kong, China; ^4^Division of Hematology & BMT Center, Queen Mary Hospital, Hong Kong, China

**Keywords:** innate lymphoid cells, graft-vs.-host disease, NK cells, T cells, hematopoietic stem cell transplantation, ILCreg

## Abstract

Allogeneic hematopoietic stem cell transplantation (Allo-HSCT) is the only curative treatment for multiple hematologic malignancies and non-malignant hematological diseases. However, graft-vs.-host disease (GVHD), one of the main complications after allo-HSCT, remains the major reason for morbidity and non-relapse mortality. Emerging evidence has demonstrated that innate lymphoid cells (ILCs) play a non-redundant role in the pathophysiology of GVHD. In this review, we will summarize previously published data regarding the role of ILCs in the pathogenesis of GVHD.

## Introduction

### Definition of ILCs

**Figure 1:**
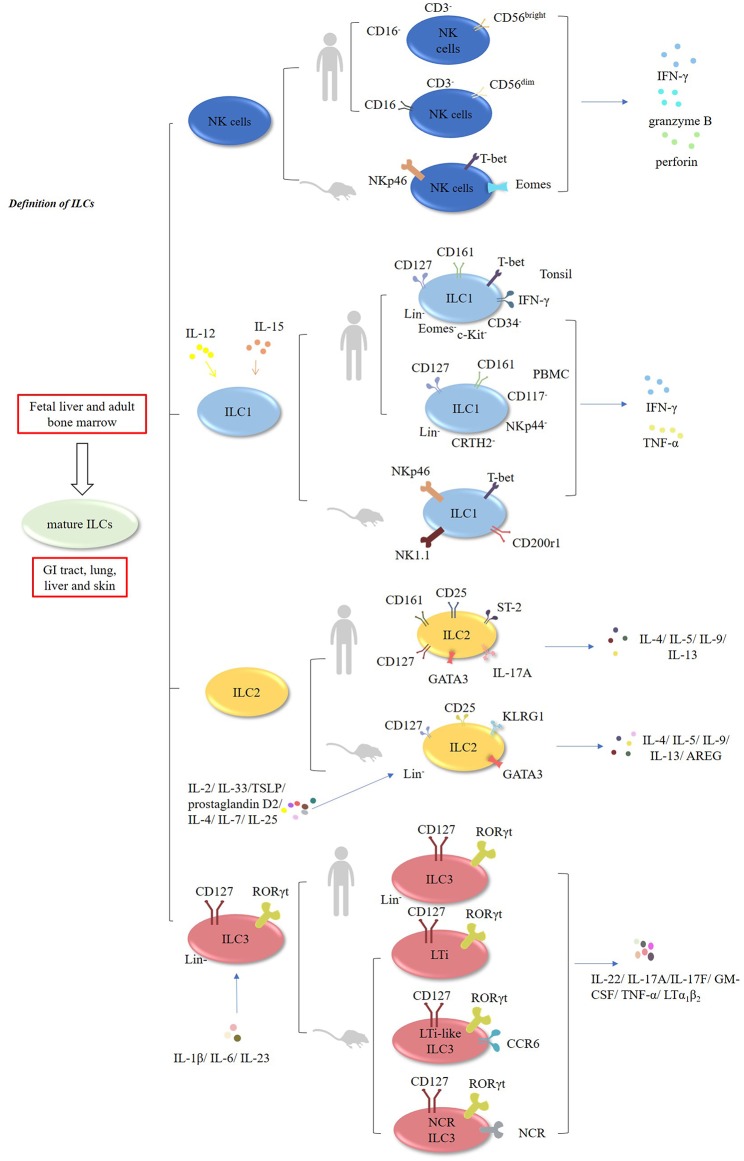
Characteristic of ILCs. ILCs encompass NK, ILC1, ILC2, and ILC3 cells. Murine and human NK cells can secrete IFN-γ, granzyme B, and perforin. In humans, NK cells have two main subsets: CD3^−^CD56^bright^CD16^−^ and CD3^−^CD56^dim^CD16^+^ cells. ILC1 cells can respond to IL-12 and IL-15, and subsequently produce IFN-γ and TNF-α. In humans, CD127^+^CD161^+^ CD34^−^ c-Kit^−^T-bet^+^ Eomes^−^ IFN-γ^+^ ILC1 cells are enriched in the tonsils. Additionally, Lin^−^CD127^+^CD161^+^ CD117^−^ NKp44^−^CRTH2^−^ ILC1 cells have been found in the human PBMCs. In mice, ILC2 cells are Lin^−^CD127^+^CD25^+^ KLRG1^+^ GATA3^high^ cells which are responsive to IL-2, IL-4, IL-7, IL-25, IL-33, TSLP, and prostaglandin D2, and subsequently produce multiple effector cytokines. In humans, ILC2 cells express GATA3, CD127, CD161, CD25, ST-2, IL-17A, and CRTH2. Both murine and human ILC3 cells are Lin^−^CD127^+^RORγt^+^. They are responsive to IL-1β, IL-6, and IL-23, and produce IL-22, IL-17A, IL-17F, GM-CSF, TNF-α, and LTα_1_β_2_.

Innate lymphoid cells (ILCs) encompass natural killer cells (NK) and ILC1, ILC2, and ILC3 cells (1–3). In contrast to T cells, these cells lack rearranged antigen receptors (1–3). It has been demonstrated that ILCs develop in the fetal liver and adult bone marrow, whereas mature ILCs are mainly enriched in the GI tract, lungs, liver, and skin (1–3). NK cells, which account for ~15% of human peripheral blood (PB) lymphocytes, exert cytolytic effects, and secrete IFN-γ, granzyme B, and perforin. In mouse, NK cells are characterized by the expression of natural killer cell p46-related protein (NKp46; also known as NCR1) receptor, and expressing transcription factors T-bet and Eomes (4–6) (Figure 1, Table 1). In humans, there are two main subsets of NK cells: CD3^−^CD56^bright^CD16^−^ and CD3^−^CD56^dim^ CD16^+^ cells (4–6) (Table 2). ILCs exhibit a cytokine repertoire that mirrors that of T helper cells. For instance, similar to Th1 cells, ILC1 cells can respond to IL-12 and IL-15 and subsequently secrete effector cytokines, such as IFN-γ and TNF-α (4–6). However, unlike NK cells, ILC1 cells do not display cytolytic effects (15). Murine ILC1 cells express Nkp46, NK1.1, T-bet, and CD200r1, but without expression of Eomes (16).

**Table 1:** Phenotype of murine ILCs.

**Marker**	**Mouse**							
	**NK**		**ILC1**		**ILC2**		**ILC3**	
CD3	–	(7)	–	(7)	–	(7)	–	(7)
CD4	–	(8)	–	(9)	–	(9)	±	(7, 9)
CD19	–	(7)	–	(7)	–	(7)	–	(7)
CD25	±	(10)	±	(10)	+	(7, 10)	±	(10)
CD45	+	(10)	+	(7, 10)	+	(7, 10)	+	(7, 10, 11)
CD49a	±	(7, 10)	+	(7, 10)	ND	–	ND	–
CD69	±	(10)	+	(10)	ND	–	ND	–
CD90	±	(10)	+	(10)	+	(10)	+	(10)
CD94	±	(10)	ND	–	±	(10)	ND	–
CD103	±	(10)	–	(10)	ND	–	ND	–
CD117	–	(10)	±	(10)	±	(10)	+	(10)
CD122	+	(10)	+	(10)	+	(10)	–	(10)
CD127	±	(10)	±	(9, 10)	+	(9, 10)	+	(7, 9, 10)
CD160	±	(10)	+	(10)	ND	–	ND	–
CD294	–	(10)	ND	–	+	(10)	ND	–
NKp46	+	(7, 10)	+	(7, 10)	–	(10)	±	(7, 10, 11)
NK1.1	+	(7, 10)	+	(7, 10)	–	(10)	±	(10)
NKG2D	+	(10)	ND	–	–	(10)	±	(10)

*ND, not determined*.

*+ positive; – negative; ± sometimes positive*.

**Table 2:** Phenotype of human ILCs.

**Marker**	**Human**							
	**NK**		**ILC1**		**ILC2**		**ILC3**	
CD1a	–	(5, 12)	–	(5, 12)	–	(5, 12)	–	(5, 12)
CD3	–	(12)	–	(5, 12)	–	(12)	–	(12)
CD4	–	(13)	±	(14)	–	(13)	±	(15)
CD7	+	ND	+	(9)	+	(9)	+	(9)
CD11c	–	(5, 12)	–	(5, 12)	–	(5, 12)	–	(5, 12)
CD14	–	(5, 12)	–	(5, 12)	–	(5, 12)	–	(5, 12)
CD16	±	(10, 15)	–	(10)	–	(10)	–	(10)
CD19	–	(5, 12)	–	(5, 12)	–	(5, 12)	–	(5, 12)
CD25	±	(10)	+	(10)	+	(10, 14, 15)	±	(10)
CD34	–	(5, 12)	–	(5, 12)	–	(5, 12)	–	(5, 12)
CD45	+	(5, 10)	+	(10)	+	(10)	+	(5, 10)
CD49a	±	(15)	±	(15)	ND	–	ND	–
CD56	+	(10, 15)	–	(5, 10)	–	(9, 10)	±	(9, 10)
CD69	±	(10)	±	(10)	ND	–	+	(5)
CD94	±	(5, 10)	–	(5, 10, 12)	–	(5, 10, 12)	–	(5, 10, 12)
CD103	±	(15)	±	(9, 15)	–	(9)	–	(9)
CD117	±	(10)	–	(10)	±	(10)	+	(10)
CD123	–	(5, 12)	–	(5, 12)	–	(5, 12)	–	(5, 12)
CD127	±	(10)	±	(10)	+	(5, 10, 13)	+	(5, 10)
CD294	–	(12)	–	(12)	+	(5, 12)	–	(12)
TCRαβ	–	(12)	–	(12)	–	(12)	–	(12)
TCRγδ	–	(12)	–	(12)	–	(12)	–	(12)
NKp46	+	(10)	–	(10)	–	(10)	±	(10)
NKp44	±	(10)	–	(10)	–	(10)	±	(5, 10)
NKp30	+	(10)	+	(10)	+	(10)	±	(10)
NK1.1	±	(10)	+	(5, 10)	+	(10)	±	(10)
NKG2D	+	(10)	ND	–	ND	–	±	(10)

*ND, not determined*.

*+ positive; – negative; ± sometimes positive*.

In humans, CD127^+^CD161^+^CD34^−^ c-Kit^−^ T-bet^+^ Eomes^−^IFN-γ^+^ILC1 cells are enriched in the tonsils (15–17). Interestingly, Lin^−^CD127^+^CD161^+^CD117^−^NKp44^−^CRTH2^−^ ILC1 cells have been found in the PBMCs of healthy individuals and atopic dermatitis (AD) patients (15–18).

ILC2 cells are defined as Lin^−^CD127^+^CD25^+^KLRG1^+^ GATA3^high^ cells in mice. These cells are responsive to multiple cytokines, including IL-2, IL-4, IL-7, IL-25, IL-33, TSLP, and prostaglandin D2, and subsequently produce Th2-type cytokines, such as IL4, IL-5, IL-9, IL-13, and amphiregulin (AREG) (1, 13, 19–26). In humans, ILC2 cells express GATA3, CD127, CD161, CD25, ST-2, IL-17A, and chemo-attractant receptor-homologous molecule expressed on Th2 lymphocytes (CRTH2) (1, 13, 15).

Both murine and human ILC3 cells are identified as Lin^−^CD127^+^RORγt^+^ cells (15). Mouse ILC3 cells consists of three subsets: lymphoid tissue-inducer cells (LTi), LTi-like CCR6-expressing ILC3 cells and NCR-expressing ILC3 cells (NCR^+^ILC3) (1, 15). Similar to Th17 cells, they are poised to respond to the stimulation by IL-1β, IL-6, and IL-23 and subsequently produce effector cytokines, such as IL-22, IL-17A, IL-17F, GM-CSF, TNF-α, and LTα_1_β_2_ (1, 15, 27–29).

NK cells are critical players in controlling intracellular bacterial and tumor surveillance (1, 15, 30). ILC1 cells are capable of controlling intracellular pathogens, whereas ILC2 cells have the capacity to limit extracellular parasitic worm infections, promote epithelial repair, and maintain mucosal tissue homeostasis. Notably, ILC2 cells are associated with chronic diseases such as pulmonary fibrosis, hepatic fibrosis, and atopic dermatitis (1, 2, 15, 30). NCR^+^ILC3 cells are the most prevalent ILC3 subset in the intestine, whereas LTi-like ILC3 cells are mainly localized in the colon and lymphoid tissues (2, 30–32). ILC3 cells are key contributors to tissue repair and protect mucosal barriers against infection by extracellular bacterial and fungi (1, 2, 30–32).

### Generation, Transcription, and Plasticity of ILCs

**Figure 2:**
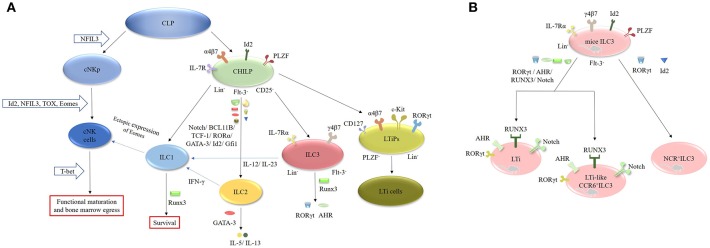
Generation, transcription and plasticity of ILCs. **(A)** ILCs originate from CLPs, which subsequently differentiate into CHILPs and cNKps. cNKps can generate cNK cells. The development of cNK cells requires Id2, NFIL3, TOX, and Eomes. Its functional maturation and bone marrow egress of these cells requires T-bet. ILC1 cells arise from Id2^+^PLZF^+^CHILP progenitor cells. The development of ILC2 cells requires Id2, GATA-3, RORα, TCF-1, BCL11B, and Notch. ILC1 cells can be converted into NK cells after ectopic expression of Eomes. IL-12 can endow ILC2 cells with ILC1 features by secreting IFN-γ, whereas IL-12 and IL-23 can induce the transition of ILC3 cells into ILC1 cells. The development of ILC2 cells requires Id2, GATA-3, RORα, TCF-1, BCL11B, and Notch. RUNX3 is necessary for the expression of RORγt and AHR in ILC3 cells. **(B)** The development of murine LTi and LTi-like ILC3 cells requires the expression of RORγt, AHR, RUNX3, and Notch, while the development of NCR^+^ILC3 cells need RORγt and Id2.

ILCs originate from common lymphoid progenitors (CLPs), which subsequently differentiate into two different lineages: the common helper-like innate lymphocyte progenitors (CHILPs) and the conventional natural killer cell progenitors (cNKps) (Figure 2). However, CHILPs are a heterogeneous population consisting of innate lymphoid cell precursors (ILCPs) and lymphoid tissue-inducer precursors (LTiPs) (33, 34). CHILPs are defined as Lin^−^IL-7R^+^Flt-3^−^α4β7^+^CD25^−^ Id2^high^PLZF^+^ cells and can give rise to ILC1, ILC2, ILC3, and LTi cells but not cNK cells (30, 33, 35). ILCPs are designated as Lin^−^CD127^+^α4β7^+^PLZF^+^ cells and can produce all ILC lineages (33). LTiPs are the precursors of LTi cells and are defined as Lin^−^CD127^+^α4β7^+^c-Kit^+^ RORγt^+^PLZF^−^ cells (33). cNKps can generate cNK cells and are unable to give rise to ILC2 and ILC3 cells. The development of cNK cells requires inhibitor of DNA binding 2 (Id2) (36–38), nuclear factor interleukin 3 (NFIL3) (39–42), thymocyte selection-associated high-mobility group box protein (TOX) (43, 44) and Eomesodermin (Eomes) (45, 46). However, the functional maturation and bone marrow egress of these cells requires T-bet (45–48). NFIL3 is involved in the development of bone marrow-derived NK cells from CLPs under homeostatic conditions and is necessary for the formation of splenic and thymic NK cells (39–42). Unlike cNK cells, ILC1 cells arise from Id2^+^PLZF^+^CHILP progenitor cells (49). Interestingly, the development of ILC2 cells requires Id2 (36, 37), GATA-binding protein 3 (GATA-3) (50–52), RORα (53), transcription factor 1 (TCF-1) (54–56), BCL11B (57, 58), and Notch (59, 60). GATA-3 is crucial for the secretion of effector cytokines, such as IL-5 and IL-13, by mature ILC2 cells (50–52, 61). In addition, Gfi1 can promote the development of ILC2 cells and control their responsiveness during infection by *Nippostrongylus brasiliensis* and protect against allergen-induced lung inflammation (62). Runx3 is another key factor in the differentiation of ILC1 and ILC3 cells. It controls the survival of ILC1 cells and is necessary for the expression of RORγt and AHR in ILC3 cells (7, 63).

ILC3 cells differentiate from Lin^−^IL-7Rα^+^Flt3^−^γ4β7^+^ fetal liver progenitors and express Id2 and RORγt in mice (1, 37). The development of murine LTi cells and LTi-like ILC3 cells requires the expression of RORγt, the aryl hydrocarbon receptor (AHR), RUNX3 and Notch (1, 2, 37, 64). The AHR seems to be involved in the expansion of CCR6^−/low^ILC3 cells (65–68). AHR^−/−^ mice exhibit a decrease in CCR6^−/low^ILC3 cells without alteration in the CCR6^+^ILC3 population. Furthermore, T-bet controls the fate and function of CCR6^−^RORγt^+^ILCs. Postnatal CCR6^−^RORγt^+^ILCs upregulate T-bet, which is modulated by the commensal microbiota. Tbx21^−/−^ mice exhibit normal development of CCR6^−^RORγt^+^ cells, but they fail to differentiate into NKp46^+^RORγt^+^ ILCs, suggesting that T-bet is necessary for the differentiation of NKp46^+^RORγt^+^ ILCs in mice (8, 69). Additionally, the IL-1β/IL-1R/MyD88 pathway controls the production of IL-22 by NKp46^+^RORγt^+^ILCs in the small intestine (SI) of mice (70). In contrast to mice, both human Lin^−^CD34^+^CD45RA^+^CD117^+^IL-1R^+^RORγt^+^ cells and stage 2 IL-1R^+^ cells in secondary lymphoid tissues (SLT) can differentiate into nearly all ILC populations including NK cells (71). Collectively, these results demonstrate that the development of ILCs is not dependent on a single “master regulator” but on a complex network of transcription factors (TFs) (1, 15, 31). Interestingly, recent studies have focused on the plasticity of ILCs. For instance, ILC1 cells can be converted into NK cells after ectopic expression of Eomes (31, 48). IL-12 can endow ILC2 cells with ILC1 features by secreting IFN-γ (60, 72), whereas IL-12 and IL-23 can induce the transition of ILC3 cells into ILC1 cells (60, 73, 74). Furthermore, dermal NCR^−^ILC3 cells can be transformed into NCR^+^ ILC3 cells in the presence of IL-1β and IL-23 *in vitro* (42, 75–77).

### Localization and Migration of ILCs

NK cells are mainly located in the bone marrow, lymph nodes, spleen, lungs, and liver, whereas ILC1 cells mainly reside in the intestinal intraepithelia (IE) (2, 78, 79). ILC2 cells are located in the lungs and lamina propria of the small intestine (SI) and skin, whereas ILC3 cells are predominantly located in the lamina propria, Peyer's patches and lymphoid follicles of the small intestine (78, 79).

It is generally considered that fetal liver and bone marrow are the “factories” where ILC subsets are generated (1, 2). However, a report by Gasteiger et al. have indicated that the vast majority of ILCs in both lymphoid and non-mymphoid organs are long-lived tissue-resident under steady state (80). Another elegant study by Di Santo JP's lab has proposed a model of “ILC-poiesis” and provided a mechanism by which tissue ILCs could be replenished from blood ILCPs in response to steady-state losses and under the circumstance of infection and inflammation (81–83).

Recently, increasing evidence has indicated that ILC1 and ILC3 cells can migrate into SLTs, depending on integrins and chemo-attractant receptors, whereas the migration of ILC2 cells from hematopoietic sites to target tissues is independent of the aforementioned receptors.

It has been indicated that the migration of NK cells to LNs via high endothelial cells (HEVs) might be mediated by CCR7 or CXCR3. The migration of ILC1 and ILC3 cells to SLTs occurs in a CCR7-dependent manner (84, 85). ILC2 cells, located in the bone marrow, spleen as well as mesenteric lymph nodes, constitutively express CCR9 and α4β7, rather than the RA-dependent homing receptor (79, 84). The migration of LTi-ILC3 cells to lymphoid follicles and the spleen marginal zone is regulated by the CXCL13-CXCR5 axis (86). Notably, trafficking receptor switches play a crucial role in the migration of ILCs. For instance, activation of spleen ILC3 cells induces upregulation of CCR9 and α4β7 with concomitant downregulation of CCR7 in the presence of IL-7 and *all-trans* retinoic acid (RA) and prompts the migration of these cells to the intestine (84, 87, 88).

### ILCs and GVHD

Allogeneic hematopoietic stem cell transplantation (Allo-HSCT) is the most powerful therapy for hematologic malignancies and a majority of non-malignant hematological diseases. One of the major barriers to the efficacy of allo-HSCT is the occurrence of GVHD. Radiotherapy/chemotherapy induction regimens damage epithelia, especially the intestinal mucosa, in recipients, followed by the translocation of commensal microbiotas from the GI tract into the peripheral blood. Subsequent activation of adaptive immunity promotes the occurrence of aGVHD (89–94).

#### The Role of Donor-Derived ILCs in GVHD

The role of NK cells in the pathogenesis of GVHD seems to be controversial (95, 96). Early studies indicated that target organs, such as the skin, liver, and GI tract, in HSCT recipients with aGVHD were infiltrated with NK cells, suggesting that NK cells might promote the development of GVHD (97–99). In accordance, administration of NK cell depleting antibodies against GM1 or NK1.1 significantly mitigated GVHD in murine models (100, 101). Cooley et al. have demonstrated that, in unrelated HSCT, increased production of IFN-γ by NK cells has correlated with more aGVHD, and decreased KIR expression has associated with inferior survival of patients, suggesting that NK cells might promote GVHD via secretion of inflammatory cytokines such as IFN-γ and TNF-α (102).

Recently, a first-in-human trial of adoptive transfer of donor-derived IL-15/4-1BBL -activated NK cells was conducted in an HLA-matched, T-cell-depleted non-myeloablative peripheral blood stem cell transplantation (103). In this clinical trial, five of nine transplant recipients experienced acute GVHD, with grade 4 GVHD in three patients. Together, the aforementioned studies seem to support the notion that NK cells promote GVHD. However, contradictory results were obtained from other studies where adoptive transfer of donor-derived NK cells into HSCT recipients can prevent the occurrence of GVHD in mouse and humans (104–107). In an MHC mismatched murine model (BALB/c → C57BL6), IL-2–activated donor-derived NK cells were administered with allogeneic bone marrow cells and splenocytes (104). Mice receiving pre-activated donor-derived NK cells significantly delayed the onset of GVHD and prolonged the survival of mice. Consistently, these mice exhibited no infiltration of inflammatory cells with normal structure of gut (104). In accordance, another animal study by Song et al. has shown that single infusion of IL-12/IL-18- pre-activated donor NK cells one day 0 after HSCT has mitigated severe or mild aGVHD, and enhanced GVL effects (108).

In line with animal data, clinical results from a phase 1 clinical trial have shown that the infusion of high doses of *ex vivo*-membrane-bound interleukin 21(mbIL-21) expanded donor-derived NK cells is safe without adverse effects, without increased GVHD or high mortality (109). Therefore, early infusion of pre-activated donor-derived NK cells has the potential of prevention of GVHD. However, it should be taken into account that different strategy for the activation of donor-derived NK cells might bring different outcomes. Other important issues that should be considered are the infusion timing of NK cells, MHC/HLA matching degree between donors and recipients as well as the pretreatment strategy before HSCT.

Interestingly, NK cells can alleviate cGVHD by directly constraining recipient minor histocompatibility Ag (mHA)-triggered proliferation of donor-derived CD4^+^ T cells in a Fas-dependent manner (110). Evidence from Ruggeri L's report has indicated that the KIR ligand incompatibility between donor and recipient might endow donor-derived NK cells to prevent the occurrence of GVHD, via direct depletion of recipient-derived antigen-presenting cells (APCs) (107). Clinical investigation on the early NK cell reconsitution in 82 patients following T cell-depleted allo-SCT have shown that NK cell number at day 14 after HSCT was inversely correlated with the incidence of grade II-IV aGVHD (111). Mechanistically, NK cells at day 14 produced high levels of IL-10 and showed upregulation of gene transcript of IL-10 compared with healthy individuals, suggesting that the regulatory phenotype might enable NK cells to suppress the development of GVHD (111).

**Figure 3:**
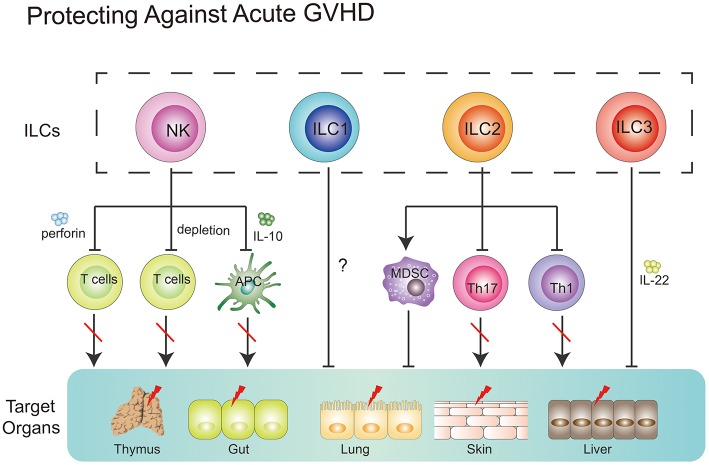
Role of ILCs in GVHD. NK cells can suppress GVHD via three main mechanisms, including direct lysing of activated T cells, indirect inhibition of T cell proliferation through depleting host APCs and production of suppressive cytokines, such as IL-10. ILC1 cells might migrate to the skin and alleviate cutaneous GVHD. Intravenous infusion of donor-derived ILC2 cells into ongoing GVHD mice can reduce the production of Th1 and Th17 cells while increasing the number of MDSCs via secreting IL-13. ILC3 cells play a protective role in GVHD. Recipient-derived ILC3 cells can alleviate pretreatment regimen-induced GI tract lesion via secretion of IL-22. Furthermore, these ILC3 cells can improve thymopoiesis in the hosts after HSCT.

Together, NK cells could prevent GVHD via (1) direct lysing of activated T cells; (2) indirect inhibition of T cell proliferation through depleting host APCs; (3) production of suppressive cytokines, such as IL-10 (Figure 3).

Only one clinical study by Munneke et al. have tried to elucidate the role of ILC1s in GVHD after HSCT (12). In the study, patients without developing aGVHD diplayed increased proportions of skin-homing donor-derived ILC1s. Notably, following transplantation, patients with more severe GVHD exhibited fewer circulating ILC1s in PB, compared with healthy controls. Mobilization of ILC1s seemed to be associated with increased expression of CD69, CLA, and CCR10 which correlated with less severe progression of GVHD (12). However, the functionality of these aforementioned ILC1s was not determined in this study. Further question is whether skin-homing ILC1s alone can prevent the occurrence of GVHD? As we know, multiple organs, including GI tract, skin, lung, liver, and mouth, in recipients are targeted in GVHD, while ILC1s-expressing CLA and CCR10, which are skin homing markers, might only traffic to the skin. Therefore, further experiments where direct infusion of ILC1s into recipients with GVHD need to be taken and will be beneficial to the understanding of the role of ILC1s in the prevention of GVHD.

It has been shown that ILC2 cells in the lower GI tract but not in the lung are sensitive to conditioning treatment and exhibit a limited repopulation ability from donor bone marrow (112). Remarkably, a single infusion of donor-derived ILC2 cells at day 7 post-HSCT was shown to remain effective at reducing the severity and mortality of ongoing aGVHD in murine model. Intravenously infused ILC2 cells migrated to the GI tract, produced Th2 cytokines, limited inflammatory Th1 and Th17 cells, and induced myeloid-derived suppressor cells (MDSCs). IL-13 produced by ILC2 cells seemed to be involved in this process. Importantly, infusion of donor ILC2 cells did not affect the beneficial graft-vs.-leukemia (GVL) effect (106). Collectively, these data indicate that intravenously infused donor-derived ILC2 cells have the capacity to alleviate ongoing aGVHD without affecting the beneficial GVL effect in murine models (112). However, several questions still require further elucidation. For instance, how do intravenously infused donor-derived ILC2 cells migrate to the GI tract in the context of GVHD? Why do these cells not migrate to the lungs of recipients? Furthermore, how do these cells survive during the migration process? All these questions require further investigation.

#### The Role of Recipient-Derived ILCs in GVHD

An increasing body of evidence has indicated that ILC3 cells have the capacity to promote tissue repair. Under homeostatic circumstances, ILC3 cells can respond to environmental signals and maintain tissue homeostasis. In contrast, abnormal signals from infection or tissue damage can activate the ILC3 response (9, 113, 114). Therefore, in GVHD conditions, induction of regimen-induced tissue damage might cause a dysregulated ILC3 response.

In an animal model, a deficiency in recipient-derived IL-22 was shown to significantly increase the severity and mortality of GVHD (113). Furthermore, pretransplantation conditions increased the intestinal expression of IL-22 in recipients, which was mainly produced by recipient-derived CD45^+^CD3^−^RORγt^+^NKp46^−^IL-7Rα^+^ CCR6^+^ ILCs. In accordance, IL-22 deficiency resulted in more severe epithelial damage during aGVHD and significant loss of intestinal stem cells. Taken together, these data suggest that loss of tissue-protective IL-22-producing ILCs in the intestines of recipients might be a pathological factor responsible for the GI tract lesions observed in aGVHD (113).

Recent work has shed light on the correlation between thymopoiesis and GVHD. Mice with GVHD after allo-HSCT exhibited a loss of intrathymic ILC3s, decreased intrathymic levels of IL-22 and impaired recovery of thymopoiesis. Not surprisingly, IL-22^−/−^ mice that underwent transplantation showed an increased severity of GVHD-associated thymic injury. IL-22 receptor^−/−^ recipient mice that underwent transplantation displayed increased numbers of cortical and medullary thymic epithelial cells (TECs). In accordance, administration of exogenous IL-22 after transplantation improved thymopoiesis and promoted the development of new thymus-derived peripheral T cells (115, 116). These findings encourage researchers to uncover what actually occurs after loss of ILC3s in the hosts induced by an induction regimen.

## Concluding Remarks

Although studies on ILCs have become a focus of research in recent years, the precise role of ILCs in the pathogenesis of GVHD remains elusive. Many questions remain to be answered in the future. For instance, what is the precise role of ILC1 cells in the pathology of GVHD? Can intravenous infusion of ILC3 cells alleviate ongoing GVHD? Lastly, how do these cells migrate to the GI tract in recipients after intravenous transfer? How about the clinical application of ILC2 for the treatment of GVHD? A recent study identified a cell population–ILCregs (117). Like Tregs, ILCregs have the suppressive ability to curb ILCs. Therefore, the question remains whether ILCregs play a role in the pathogenesis of GVHD? Additionally, what is the interaction between ILCs and ILCregs at the onset of GVHD? These questions require further elucidation in future work.

## Author Contributions

LS drafted the manuscript. AL, FZ, and QZ revised the manuscript. SP completed the figures. MJ and LC did the language editing. All authors read this manuscript and approved its submission.

### Conflict of Interest Statement

The authors declare that the research was conducted in the absence of any commercial or financial relationships that could be construed as a potential conflict of interest.

## References

[1] Spits H Cupedo T. Innate lymphoid cells: emerging insights in development, lineage relationships, and function. Annu Rev Immunol. (2012) 30:647–75. 10.1146/annurev-immunol-020711-075053 22224763

[2] Kim CH Hashimoto-Hill S Kim M. Migration and Tissue Tropism of Innate Lymphoid Cells. Trends Immunol. (2016) 37:68–79. 10.1016/j.it.2015.11.003 26708278 PMC4744800

[3] Spits H Di Santo JP. The expanding family of innate lymphoid cells: regulators and effectors of immunity and tissue remodeling. Nat Immunol. (2011) 12:21–7. 10.1038/ni.1962 21113163

[4] Fuchs A Vermi W Lee JS Lonardi S Gilfillan S Newberry RD. Intraepithelial type 1 innate lymphoid cells are a unique subset of IL-12- and IL-15-responsive IFN-γ-producing cells. Immunity. (2013) 38:769–81. 10.1016/j.immuni.2013.02.010 23453631 PMC3634355

[5] Bernink JH Peters CP Munneke M te Velde AA Meijer SL Weijer K. Human type 1 innate lymphoid cells accumulate in inflamed mucosal tissues. Nat Immunol. (2013) 14, 221–9. 10.1038/ni.2534 23334791

[6] Mortha A Burrows K. Cytokine networks between innate lymphoid cells and myeloid cells. Front Immunol. (2018) 9:191. 10.3389/fimmu.2018.00191 29467768 PMC5808287

[7] Ebihara T Song C Ryu SH Plougastel-Douglas B Yang L Levanon D. Runx3 specifies lineage commitment of innate lymphoid cells. Nat Immunol. (2015) 16:1124–33. 10.1038/ni.3272 26414766 PMC4618046

[8] Klose CS Kiss EA Schwierzeck V Ebert K Hoyler T d'Hargues Y. A T-bet gradient controls the fate and function of CCR6-RORγt+ innate lymphoid cells. Nature. (2013) 494:261–5. 10.1038/nature11813 23334414

[9] Konya V Mjösberg J. Innate lymphoid cells in graft-versus-host disease. Am J Transplant. (2015) 15:2795–801. 10.1111/ajt.13394 26228632 PMC4973689

[10] Vivier E Artis D Colonna M Diefenbach A Di Santo JP Eberl G. Innate lymphoid cells: 10 years on. Cell. (2018) 174:1054–66. 10.1016/j.cell.2018.07.017 30142344

[11] Liu B Ye B Zhu X Huang G Yang L Zhu P. IL-7Rα glutamylation and activation of transcription factor Sall3 promote group 3 ILC development. Nat Commun. (2017) 8:231. 10.1038/s41467-017-00235-x 28794449 PMC5550436

[12] Munneke JM Björklund AT Mjösberg JM Garming-Legert K Bernink JH Blom B. Activated innate lymphoid cells are associated with a reduced susceptibility to graft-versus-host disease. Blood. (2014) 124:812–21. 10.1182/blood-2013-11-536888 24855210

[13] Mjösberg JM Trifari S Crellin NK Peters CP van Drunen CM Piet B. Human IL-25- and IL-33-responsive type 2 innate lymphoid cells are defined by expression of CRTH2 and CD161. Nat Immunol. (2011) 12:1055–62. 10.1038/ni.2104 21909091

[14] Tait Wojno ED Artis D. Emerging concepts and future challenges in innate lymphoid cell biology. J Exp Med. (2016) 213:2229–48. 10.1084/jem.20160525 27811053 PMC5068238

[15] Montaldo E Vacca P Vitale C Moretta F Locatelli F Mingari MC. Human innate lymphoid cells. Immunol Lett. (2016).179:2–8. 10.1016/j.imlet.2016.01.007 26844414

[16] Adams NM Sun JC. Spatial and temporal coordination of antiviral responses by group 1 ILCs. Immunol Rev. (2018) 286:23–36. 10.1111/imr.12710 30294970 PMC6178831

[17] Colonna M. Innate lymphoid cells: diversity, plasticity, and unique functions in immunity. Immunity. (2018) 48:1104–17. 10.1016/j.immuni2018.05.013 29924976 PMC6344351

[18] Villanova F Flutter B Tosi I Grys K Sreeneebus H Perera GK. Characterization of innate lymphoid cells in human skin and blood demonstrates increase of NKp44+ ILC3 in psoriasis. J Invest Dermatol. (2014) 134:1–8. 10.1038/jid.2013.477 24352038 PMC3961476

[19] Kim BS Siracusa MC Saenz SA Noti M Monticelli LA Sonnenberg GF. TSLP elicits IL-33-independent innate lymphoid cell responses to promote skin inflammation. Sci Transl Med. (2013) 5:170ra16. 10.1126/scitranslmed.3005374 23363980 PMC3637661

[20] Imai Y Yasuda K Sakaguchi Y Haneda T Mizutani H Yoshimoto T. Skin-specific expression of IL-33 activates group 2 innate lymphoid cells and elicits atopic dermatitis-like inflammation in mice. Proc Natl Acad Sci USA. (2013) 110:13921–6. 10.1073/pnas.1307321110 23918359 PMC3752227

[21] Xue L Salimi M Panse I Mjosberg JM McKenzie AN Spits H. Prostaglandin D2 activates group 2 innate lymphoid cells through chemoattractant receptor-homologous molecule expressed on TH2 cells. J Allergy Clin Immunol. (2014) 133:1184–94. 10.1016/j.jaci.2013.10.056 24388011 PMC3979107

[22] Doherty TA Khorram N Lund S Mehta AK Croft M Broide DH. Lung type 2 innate lymphoid cells express cysteinyl leukotriene receptor 1, which regulates TH2 cytokine production. J Allergy Clin Immunol. (2013) 132:205–13. 10.1016/j.jaci.2013.03.048 23688412 PMC3704056

[23] Karta MR Broide DH Doherty TA. Insights into group 2 innate lymphoid cells in human airway disease. Curr Allergy Asthma Rep. (2016) 16:8. 10.1007/s11882-015-0581-6 26746844 PMC5026503

[24] Monticelli LA Osborne LC Noti M Tran SV Zaiss DM Artis D. IL-33 promotes an innate immune pathway of intestinal tissue protection dependent on amphiregulin-EGFR interactions. Proc Natl Acad Sci USA. (2015) 112:10762–7. 10.1073/pnas.1509070112 26243875 PMC4553775

[25] Monticelli LA Sonnenberg GF Abt MC Alenghat T Ziegler CG Doering TA. Innate lymphoid cells promote lung-tissue homeostasis after infection with influenza virus. Nat Immunol. (2011) 12:1045–54. 10.1031/ni.2131 21946417 PMC3320042

[26] Zaiss DMW Gause WC Osborne LC Artis D. Emerging functions of amphiregulin in orchestrating immunity, inflammation, and tissue repair. Immunity. (2015) 42:216–26. 10.1016/j.immuni.2015.01.020 25692699 PMC4792035

[27] Kruglov AA Grivennikov SI Kuprash DV Winsauer C Prepens S Seleznik GM. Nonredundant function of soluble LTα3 produced by innate lymphoid cells in intestinal homeostasis. Science. (2013) 342:1243–6. 10.1126/science.1243364 24311691

[28] Cording S Medvedovic J Cherrier M Eberl G. Development and regulation of RORγt(+) innate lymphoid cells. FEBS Lett. (2014) 588:4176–81. 10.1016/j.febslet.2014.03.034 24681095

[29] Mortha A Chudnovskiy A Hashimoto D Bogunovic M Spencer SP Belkaid Y. Microbiota-dependent crosstalk between macrophages and ILC3 promotes intestinal homeostasis. Science. (2014) 343:1249288. 10.1126/science.1249288 24625929 PMC4291125

[30] Zook EC Kee BL. Development of innate lymphoid cells. Nat Immunol. (2016) 17:775–82. 10.1038/ni.3481 27328007

[31] Serafini N Vosshenrich CA Di Santo JP. Transcriptional regulation of innate lymphoid cell fate. Nat Rev Immunol. (2015) 15:415–28. 10.1038/nri3855 26065585

[32] Lim AI Verrier T Vosshenrich CA Di Santo JP. Developmental options and functional plasticity of innate lymphoid cells. Curr Opin Immunol. (2017) 44:61–8. 10.1016/j.coi.2017.03.010 28359987

[33] Ishizuka IE Constantinides MG Gudjonson H Bendelac A. The innate lymphoid cell precursor. Annu Rev Immunol. (2016) 34:299–316. 10.1146/annurev-immunol-041015-055549 27168240

[34] Klose CSN Flach M Möhle L Rogell L Hoyler T Ebert K. Differentiation of type 1 ILCs from a common progenitor to all helper-like innate lymphoid cell lineages. Cell. (2014) 157:340–56. 10.1016/j.cell.2014.03.030 24725403

[35] Constantinides MG McDonald BD Verhoef PA Bendelac A. A committed precursor to innate lymphoid cells. Nature. (2014) 508:397–401. 10.1038/nature13047 24509713 PMC4003507

[36] Guo X Liang Y Zhang Y Lasorella A Kee BL Fu YX. Innate lymphoid cells control early colonization resistance against intestinal pathogens through ID2-dependent regulation of the microbiota. Immunity. (2015) 42:731–43. 10.1016/j.immuni.2015.03.012 25902484 PMC4725053

[37] Cherrier M Sawa S Eberl G. Notch, Id2, and RORgammat sequentially orchestrate the fetal development of lymphoid tissue inducer cells. J Exp Med. (2012) 209:729–40. 10.1084/jem.20111594 22430492 PMC3328368

[38] Geiger TL Sun JC. Development and maturation of natural killer cells. Curr Opin Immunol. (2016) 39:82–9. 10.1016/j.coi.2016.01.007 26845614 PMC4801705

[39] Kamizono S Duncan GS Seidel MG Morimoto A Hamada K Grosveld G. Nfil3/E4bp4 is required for the development and maturation of NK cells *in vivo*. J Exp Med. (2009) 206:2977–86. 10.1084/jem.20092176 19995955 PMC2806474

[40] Xu W Domingues RG Fonseca-Pereira D Ferreira M Ribeiro H Lopez-Lastra S. NFIL3 orchestrates the emergence of common helper innate lymphoid cell precursors. Cell Rep. (2015) 10:2043–54. 10.1016/j.celrep.2015.02.057 25801035

[41] Kostrzewski T Borg AJ Meng Y Filipovic I Male V Wack A. Multiple levels of control determine how E4bp4/Nfil3 regulates NK cell development. J Immunol. (2018) 200:1370–81. 10.4049/jimmunol.1700981 29311361 PMC5812440

[42] Sciumè G Shih HY Mikami Y O'Shea JJ. Epigenomic views of innate lymphoid cells. Front Immunol. (2017) 8:1579. 10.3389/fimmu.2017.01579 29250060 PMC5715337

[43] Vong QP Leung WH Houston J Li Y Rooney B Holladay M. TOX2 regulates human natural killer cell development by controlling T-BET expression. Blood. (2014) 124:3905–13. 10.1182/blood-2014-06-582965 25352127 PMC4282154

[44] Mora-Velandia LM Castro-Escamilla O Méndez AG Aguilar-Flores C Velázquez-Avila M Tussié-Luna MI. A human Lin- CD123+ CD127low population endowed with ILC features and migratory capabilities contributes to immunopathological hallmarks of psoriasis. Front Immunol. (2017) 8:176. 10.3389/fimmu.2017.00176 28303135 PMC5332395

[45] Daussy C Faure F Mayol K Viel S Gasteiger G Charrier E. T-bet and Eomes instruct the development of two distinct natural killer cell lineages in the liver and in the bone marrow. J Exp Med. (2014) 211:563–77. 10.1084/jem.20131560 24516120 PMC3949572

[46] Zhang J Marotel M Fauteux-Daniel S Mathieu AL Viel S Marçais A. T-bet and Eomes govern differentiation and function of mouse and human NK cells and ILC1. Eur J Immunol. (2018) 48:738–50. 10.1002/eji.201747299 29424438

[47] Simonetta F Pradier A Roosnek E. T-bet and eomesodermin in NK cell development, maturation, and function. Front Immunol. (2016) 7:241. 10.3389/fimmu.2016.00241 27379101 PMC4913100

[48] Pikovskaya O Chaix J Rothman NJ Collins A Chen YH Scipi-oni A M. Cutting edge: eomesodermin is sufficient to direct type 1 innate lymphocyte development into the conventional NK lineage. J Immunol. (2016) 196:1449–54. 10.4049/jimmunol.1502396 26792802 PMC4744497

[49] Constantinides MG Gudjonson H McDonald BD Ishizuka IE Verhoef PA Dinner AR. PLZF expression maps the early stages of ILC1 lineage development. Proc Natl Acad Sci USA. (2015) 112:5123–8. 10.1073/pnas.1423244112 25838284 PMC4413309

[50] Mjosberg J Bernink J Golebski K Karrich JJ Peters CP Blom B. The transcription factor GATA3 is essential for the function of human type 2 innate lymphoid cells. Immunity. (2012) 37:649–59. 10.1016/j.immuni.2012.08.015 23063330

[51] Klein Wolterink RG Serafini N van Nimwegen M Vosshenrich CA de Bruijn MJ Fonseca Pereira D. Essential, dose-dependent role for the transcription factor Gata3 in the development of IL-5+ and IL-13+ type 2 innate lymphoid cells. Proc Natl Acad Sci USA. (2013) 110:10240–5. 10.1073/pnas.1217158110 23733962 PMC3690884

[52] Walker JA McKenzie AN. Development and function of group 2 innate lymphoid cells. Curr Opin Immunol. (2013) 25:148–55. 10.1016/j.coi.2013.02.010 23562755 PMC3776222

[53] Wong SH Walker JA Jolin HE Drynan LF Hams E Camelo A. Transcription factor RORalpha is critical for nuocyte development. Nat Immunol. (2012) 13:229–36. 10.1038/ni.2208 22267218 PMC3343633

[54] Mielke LA Groom JR Rankin LC Seillet C Masson F Putoczki T. TCF-1 controls ILC2 and NKp46+RORγt+ innate lymphocyte differentiation and protection in intestinal inflammation. J Immunol. (2013) 191:4383–91. 10.4049/jimmunol.1301228 24038093

[55] Ishizuka IE Chea S Gudjonson H Constantinides MG Dinner AR Bendelac A. Single-cell analysis defines the divergence between the innate lymphoid cell lineage and lymphoid tissue-inducer cell lineage. Nat Immunol. (2016) 17:269–76. 10.1038/ni.3344 26779601 PMC4755916

[56] Seillet C Mielke LA Amann-Zalcenstein DB Su S Gao J Almeida FF. Deciphering the innate lymphoid cell transcriptional program. Cell Rep. (2016) 17:436–47. 10.1016/j.celrep.09.025 27705792

[57] Yu Y Wang C Clare S Wang J Lee SC Brandt C. The transcription factor Bcl11b is specifically expressed in group 2 innate lymphoid cells and is essential for their development. J Exp Med. (2015) 212:865–74. 10.1084/jem.20142318 25964371 PMC4451136

[58] Califano D Cho JJ Uddin MN Lorentsen KJ Yang Q Bhandoola A. Transcription factor Bcl11b controls identity and function of mature type 2 innate lymphoid cells. Immunity. (2015) 43:354–68. 10.1016/j.immuni.2015.07.005 26231117 PMC4657441

[59] Zhang K Xu X Pasha MA Siebel CW Costello A Haczku A. Cutting edge: notch signaling promotes the plasticity of group-2 innate lymphoid cells. J Immunol. (2017) 198:1798–803. 10.4049/jimmunol.1601421 28115527 PMC5321819

[60] Lim AW McKenzie AN. Deciphering the transcriptional switches of innate lymphoid cell programming: the right factors at the right time. Genes Immun. (2015) 16:177–86. 10.1038/gene.2014.83 25611557 PMC4409422

[61] Tindemans I Serafini N Di Santo JP Hendriks RW. GATA-3 function in innate and adaptive immunity. Immunity. (2014) 41:191–206. 10.1016/j.immuni.2014.06.006 25148023

[62] Spooner CJ Lesch J Yan D Khan AA Abbas A Ramirez-Carrozzi V. Specification of type 2 innate lymphocytes by the transcriptional determinant Gfi1. Nat Immunol. (2013) 14:1229–36. 10.1038/ni.2743 24141388

[63] Harly C Cam M Kaye J Bhandoola A. Development and differentiation of early innate lymphoid progenitors. J Exp Med. (2018) 215:249–62. 10.1084/jem.20170832 29183988 PMC5748853

[64] Rankin LC Groom JR Chopin M Herold MJ Walker JA Mielke LA. The transcription factor T-bet is essential for the development of NKp46+ innate lymphocytes via the Notch pathway. Nat Immunol. (2013) 14:389–95. 10.1038/ni.2545 23455676 PMC4076532

[65] Kiss EA Vonarbourg C Kopfmann S Hobeika E Finke D Esser C. Natural aryl hydrocarbon receptor ligands control organogenesis of intestinal lymphoid follicles. Science. (2011) 334:1561–5. 10.1126/science.1214914 22033518

[66] Qiu J Heller JJ Guo X Chen ZM Fish K Fu YX. The aryl hydrocarbon receptor regulates gut immunity through modulation of innate lymphoid cells. Immunity. (2012) 36:92–104. 10.1016/j.immuni.2011.11.011 22177117 PMC3268875

[67] Qiu J Zhou L. Aryl hydrocarbon receptor promotes RORγt+ group 3 ILCs and controls intestinal immunity and inflammation. Semin Immunopathol. (2013) 35:657–70. 10.1007/s00281-013-0393-5 23975386 PMC3797199

[68] Li S Bostick JW Zhou L. Regulation of innate lymphoid cells by Aryl hydrocarbon receptor. Front Immunol. (2018) 8:1909. 10.3389/fimmu.2017.01909 29354125 PMC5760495

[69] Powell N Walker AW Stolarczyk E Canavan JB Gökmen MR Marks E. The transcription factor T-bet regulates intestinal inflammation mediated by interleukin-7 receptor+ innate lymphoid cells. Immunity. (2012) 37:674–84. 10.1016/j.immuni.2012.09.008 23063332 PMC3540260

[70] Reynders A Yessaad N Vu Manh TP Dalod M Fenis A Aubry C. Identity, regulation and *in vivo* function of gut NKp46+RORγt+ and NKp46+RORγt- lymphoid cells. EMBO J. (2011) 30:2934–47. 10.1038/emboj.2011.201 21685873 PMC3160256

[71] Scoville SD Mundy-Bosse BL Zhang MH Chen L Zhang X Keller KA. A progenitor cell expressing transcription factor RORγt generates all human innate lymphoid cell subsets. Immunity. (2016) 44:1140–50. 10.1016/j.immuni.2016.04.007 27178467 PMC4893782

[72] Lim AI Menegatti S Bustamante J Le Bourhis L Allez M Rogge L. IL-12 drives functional plasticity of human group 2 innate lymphoid cells. J Exp Med. (2016) 213:569–83. 10.1084/jem.20151750 26976630 PMC4821648

[73] Bernink JH Krabbendam L Germar K de Jong E Gronke K Kofoed-Nielsen M. Interleukin-12 and−23 control plasticity of CD127(+) Group 1 and Group 3 innate lymphoid cells in the intestinal lamina propria. Immunity. (2015) 43:146–60. 10.1016/j.immuni.2015.06.019 26187413

[74] Montaldo E Juelke K Romagnani C. Group 3 innate lymphoid cells (ILC3s): Origin, differentiation, and plasticity in humans and mice. Eur J Immunol. (2015) 45:2171–82. 10.1002/eji.201545598 26031799

[75] Teunissen MBM Munneke JM Bernink JH Spuls PI Res PCM Te Velde A. Composition of innate lymphoid cell subsets in the human skin: enrichment of NCR(+) ILC3 in lesional skin and blood of psoriasis patients. J Invest Dermatol. (2014) 134:2351–60. 10.1038/jid.2014.146 24658504

[76] Zhong C Zhu J. Transcriptional regulators dictate innate lymphoid cell fates. Protein Cell. (2017) 8:242–54. 10.1007/s13238-017-0369-7 28108952 PMC5359184

[77] Simoni Y Newell EW. Dissecting human ILC heterogeneity: more than just three subsets. Immunology. (2018) 153:297–303. 10.1111/imm.12862 29140572 PMC5795188

[78] Soriani A Stabile H Gismondi A Santoni A Bernardini G. Chemokine regulation of innate lymphoid cell tissue distribution and function. Cytokine Growth Factor Rev. (2018). 10.1016/j.cytogfr.2018.02.003 29472011

[79] Ignacio A Breda CNS Camara NOS. Innate lymphoid cells in tissue homeostasis and diseases. World J Hepatol. (2017) 9:979–89. 10.4254/wjh.v9.i23.979 28878863 PMC5569277

[80] Gasteiger G Fan X Dikiy S Lee SY Rudensky AY. Tissue residency of innate lymphoid cells in lymphoid and nonlymphoid organs. Science. (2015) 350:981–5. 10.1126/science.aac9593 26472762 PMC4720139

[81] Lim AI Li Y Lopez-Lastra S Stadhouders R Paul F Casrouge A Serafini N. Systemic human ILC precursors provide a substrate for tissue ILC differentiation. Cell. (2017) 168:1086–100.e10. 10.1016/j.cell.2017.02.021 28283063

[82] Lim AI Di Santo JP. ILC-poiesis: ensuring tissue ILC differentiation at the right place and time. Eur J Immunol. (2018) 49:11–8. 10.1002/eji.201747294 30350853

[83] Mjösberg J Mazzurana L. ILC-poiesis: making tissue ILCs from blood. Immunity. (2017) 46:344–6. 10.1016/j.immuni.2017.03.002 28329700

[84] Kim MH Taparowsky EJ Kim CH. Retinoic acid differentially regulates the migration of innate lymphoid cell subsets to the gut. Immunity. (2015) 43:107–19. 10.1016/j.immuni.2015.06.009 26141583 PMC4511719

[85] Mackley EC Houston S Marriott CL Halford EE Lucas B Cerovic V. CCR7-dependent trafficking of RORγ+ ILCs creates a unique microenvironment within mucosal draining lymph nodes. Nat Commun. (2015) 6:5862. 10.1038/ncomms6862 25575242 PMC4354100

[86] Marchesi F Martin AP Thirunarayanan N Devany E Mayer L Grisotto MG. CXCL13 expression in the gut promotes accumulation of IL-22-producing lymphoid tissue-inducer cells, and formation of isolated lymphoid follicles. Mucosal Immunol. (2009) 2:486–94. 10.1038/mi.2009.113 19741597

[87] van de Pavert SA Ferreira M Domingues RG Ribeiro H Molenaar R Moreira-Santos L. Maternal retinoids control type 3 innate lymphoid cells and set the offspring immunity. Nature. (2014) 508:123–7. 10.1038/nature13158 24670648 PMC4932833

[88] Goverse G Labao-Almeida C Ferreira M Molenaar R Wahlen S Konijn T. Vitamin A controls the presence of RORγ+ innate lymphoid cells and lymphoid tissue in the small intestine. J Immunol. (2016) 196:5148–55. 10.4049/jimmunol.1501106 27183576

[89] Zeiser R Blazar BR. Acute graft-versus-host disease - biologic process, prevention, and therapy. N Engl J Med. **(**2017) 377:2167–79. 10.1056/NEJMra1609337 29171820 PMC6034180

[90] Ferrara JL Levine JE Reddy P Holler E. Graft-versus-host disease. Lancet. **(**2009) 373:1550–61. 10.1016/S0140-6736(09)60237-3 19282026 PMC2735047

[91] Hu Y Cui Q Luo C Luo Y Shi J Huang H. A promising sword of tomorrow: human γ*δ* T cell strategies reconcile allo-HSCT complications. Blood Rev. **(**2016) 30:179–88. 10.1016/j.blre.2015.11.002 26654098

[92] Zeng D Lan F Hoffmann P Strober S. Suppression of graft-versus-host disease by naturally occurring regulatory T cells. Transplantation. (2004) 77(1 Suppl.): S9–11. 10.1097/01.TP.0000106475.38978.11 14726761

[93] Pessach I Tsirigotis P Nagler A. The gastrointestinal tract: properties and role in allogeneic hematopoietic stem cell transplantation. Expert Rev Hematol. (2017) 10:315–26. 10.1080/17474086.2017.1288566 28136133

[94] Zeiser R Socié G Blazar BR. Pathogenesis of acute graft-versus-host disease: from intestinal microbiota alterations to donor T cell activation. Br J Haematol. (2016) 175:191–207. 10.1111/bjh.14295 27619472

[95] Blazar BR MacDonald KPA Hill GR. Immune regulatory cell infusion for graft-versus-host disease prevention and therapy. Blood. (2018) 131:2651–60. 10.1182/blood-2017-11-785865 29728401 PMC6032895

[96] Simonetta F Alvarez M Negrin RS. Natural killer cells in graft-versus-host-disease after allogeneic hematopoietic cell transplantation. Front Immunol. (2017) 8:465. 10.3389/fimmu.2017.00465 28487696 PMC5403889

[97] Horn TD Haskell J. The lymphocytic infiltrate in acute cutaneous allogeneic graft-versus-host reactions lacks evidence for phenotypic restriction in donor-derived cells. J Cutan Pathol. (1998) 25:210–4. 9609140 10.1111/j.1600-0560.1998.tb01721.x

[98] Dilly SA Sloane JP. An immunohistological study of human hepatic graft-versus-host disease. Clin Exp Immunol. (1985) 62:545–53. 3910317 PMC1577456

[99] Roy J Platt JL Weisdorf DJ. The immunopathology of upper gastrointestinal acute graft-versus-host disease. Lymphoid cells and endothelial adhesion molecules. Transplantation. (1993) 55:572–8. 7681225 10.1097/00007890-199303000-00022

[100] Charley MR Mikhael A Hackett J Kumar V Bennett M. Mechanism of anti-asialo GM1 prevention of graft-vs-host disease: identification of allo-antigen activated T cells. J Invest Dermatol. (1988) 91:202–6. 3261762 10.1111/1523-1747.ep12464858

[101] Zeng D Lewis D Dejbakhsh-Jones S Lan F García-Ojeda M Sibley R. Bone marrow NK1.1(-) and NK1.1(+) T cells reciprocally regulate acute graft versus host disease. J Exp Med. (1999) 189:1073–81. 10190898 10.1084/jem.189.7.1073 PMC2193016

[102] Cooley S McCullar V Wangen R Bergemann TL Spellman S Weisdorf DJ. KIR reconstitution is altered by T cells in the graft and correlates with clinical outcomes after unrelated donor transplantation. Blood. (2005) 106:4370–6. 10.1182/blood-2005-04-1644 16131567 PMC1895239

[103] Shah NN Baird K Delbrook CP Fleisher TA Kohler ME Rampertaap S. Acute GVHD in patients receiving IL-15/4-1BBL activated NK cells following T-cell-depleted stem cell transplantation. Blood. (2015) 125:784–92. 10.1182/blood-2014-07-592881 25452614 PMC4311226

[104] Tanaka M Kobayashi S Numata A Tachibana T Takasaki H Maruta A. The impact of the dose of natural killer cells in the graft on severe acute graft-versus-host disease after unrelated bone marrow transplantation. Leuk Res. (2012) 36:699–703. 10.1016/j.leukres.2011.11.009 22172462

[105] Kim SY Lee H Han MS Shim H Eom HS Park B. Post-transplantation natural killer cell count: a predictor of acute graft-versus-host disease and survival outcomes after allogeneic hematopoietic stem cell transplantation. Clin Lymphoma Myeloma Leuk. (2016) 16:527–35.e2. 10.1016/j.clml.2016.06.013 27375156

[106] Wong PPC Kariminia A Jones D Eaves CJ Foley R Ivison S. Plerixafor effectively mobilizes CD56bright NK cells in blood, providing an allograft predicted to protect against GVHD. Blood. (2018) 131:2863–6. 10.1182/blood-2018-03-836700 29728400

[107] Ruggeri L Capanni M Urbani E Perruccio K Shlomchik WD Tosti A. Effectiveness of donor natural killer cell alloreactivity in mismatched hematopoietic transplants. Science. (2002) 295:2097–100. 10.1126/science.1068440 11896281

[108] Song Y Hu B Liu Y Jin Z Zhang Y Lin D. IL-12/IL-18- preactivated donor NK cells enhance GVL effects and mitigate GvHD after allogeneic hematopoietic stem cell transplantation. Eur J Immunol. (2018) 48:670–82. 10.1002/eji.201747177 29282719

[109] Ciurea SO Schafer JR Bassett R Denman CJ Cao K Willis D. Phase 1 clinical trial using mbIL21 *ex vivo*-expanded donor-derived NK cells after haploidentical transplantation. Blood. (2017) 130:1857–68. 10.1182/blood-2017-05-785659 28835441 PMC5649552

[110] Noval Rivas M Hazzan M Weatherly K Gaudray F Salmon I Braun MY. NK cell regulation of CD4 T cell-mediated graft-versus-host disease. J Immunol. (2010) 184:6790–8. 10.4049/jimmunol.0902598 20488796

[111] Chan YLT Zuo J Inman C Croft W Begum J Croudace J. NK cells produce high levels of IL-10 early after allogeneic stem cell transplantation and suppress development of acute GVHD. Eur J Immunol. 48:316–29. 10.1002/eji.201747134 28944953 PMC5836991

[112] Bruce DW Stefanski HE Vincent BG Dant TA Reisdorf S Bommiasamy H. Type 2 innate lymphoid cells treat and prevent acute gastrointestinal graft-versus-host disease. J Clin Invest. (2017) 127:1813–25. 10.1172/JCI91816 28375154 PMC5409787

[113] Hanash AM Dudakov JA Hua G O'Connor MH Young LF Singer NV. Interleukin-22 protects intestinal stem cells from immune-mediated tissue damage and regulates sensitivity to graft versus host disease. Immunity. (2012) 37:339–50. 10.1016/j.immuni.2012.05.028 22921121 PMC3477611

[114] Karrich JJ Cupedo T. Group 3 innate lymphoid cells in tissue damage and graft-versus-host disease pathogenesis. Curr Opin Hematol. (2016) 23:410–5. 10.1097/MOH.0000000000000262 27135976

[115] Dudakov JA Mertelsmann AM O'Connor MH Jenq RR Velardi E Young LF. Loss of thymic innate lymphoid cells leads to impaired thymopoiesis in experimental graft-versus-host disease. Blood. (2017) 130:933–42. 10.1182/blood-2017-01-762658 28607133 PMC5561900

[116] Komanduri KV. Innately interesting interactions. Blood. (2017) 130:844–5. 10.1182/blood-2017-06-791848 28818978

[117] Wang S Xia P Chen Y Qu Y Xiong Z Ye B. Regulatory innate lymphoid cells control innate intestinal inflammation. Cell. (2017) 171:201–16. 10.1016/j.cell.2017.07.027 28844693

